# Eyes and Noses

**Published:** 1890-11-08

**Authors:** 


### Eyes and Noses.

"What," some one asks the Autocrat of the Breakfast-table, "do you think of phrenology?" "Let me begin," replies the genial tyrant, "by defining a pseudo-science." We should start in somewhat the same fashion if pressed to state the exact esteem in which we hold physiognomy. That there is "something in it" we are quite convinced, but anything more definite we are not prepared to say. At the first meeting with anyone, man or woman, we receive an impression, favourable or the reverse, from their appearance. Often we cannot analyse this impression nor explain it in any way. It does not depend on beauty or its absence; there are plain faces that win us and pretty ones that repel. Perhaps, on the whole, we incline to the pretty ones; but that depends to some extent on our sex, and that of the owner of the face. But whencesoever it springs the impression remains, and though it may be that "second thoughts are best," it is equally true that "first impressions are lasting." Some of these prepossessions may be explained on sufficiently scientific grounds. Natural selection, the instinctive, unconscious desire to be the parent of something better than oneself, explains why short men insist on offering themselves to "daughters of the gods, divinely tall"; why Francis Osbaldistone, who lingers in our memory as rather a milksop, adored high-spirited, energetic Diana Vernon; why the "wooden spoon" sighs afar off for the lady senior wrangler. These things we can understand, and to some extent explain; but there is a whole host of minor preferences that baffle our reasoning powers, and we are as erratic and as obstinate as the poet who sang of Dr. Fell. Can the physiognomists help us? Can they tell why we hate Sabidius while we delight in Balbus, his kinsman, the friend of our youth? Perhaps they can.

To a certain extent we are all physiognomists. When we see Mr. William Sykes hanging about our premises we incline to lock up the spoons as a measure of precaution, and even to enter into conversation—silver speech—with the policeman on the beat. Certain types of face, as appertaining to certain mental characteristics, are fixed in the popular imagination. If Mr. Du Maurier, for example, were to present us with one of his broad-browed, short-nosed, simious scientists as the portrait of a High Church curate, we should resent it as an insult to our understanding, or at least to our preconceptions. Rightly or wrongly, we have fixed ideas about the appearance of the man of science, of business, of art, of religion, and much disillusionment fails to disillusion us. The root of this matter of physiognomy is in us, though perhaps not all the twigs, and leaves, and branches.

It may be that on such a subject the average man has no right to speak. The average man is notoriously inaccurate in things outside his own vocation. When he sees his neighbour look sallow and pale he says "liver," when perhaps it is "kidney," and he is apt to confuse consumption with pleurisy on the plea that each is "something wrong with the lungs." In physiognomy, then, he may be as far from the truth as in pathology, and may require the guidance of a wiser mind. But who shall lead him? If he falls into the hands of as grave and moderate a cicerone as Signor Mantegazza he will not go far astray—a man who is careful to put down only what is known of physiognomy, and to keep his inferences within the bounds of general knowledge. But if he trust himself to the guidance of Mrs. Mary Olmsted Stanton, then shall we see strange things. Mrs. Stanton has just published, here and in America simultaneously, "A System of Practical and Scientific Physiognomy; or How to Read Faces; being a Manual of Instruction in the Knowledge of the Human Physiognomy and Organism, embracing the Discoveries of Located Signs of Character in the Body and Face, as shown by the Five Natural Divisions of the Countenance." That is all. The work is "affectionately dedicated to the lovers of science, to the earnest and enthusiastic searchers for truth throughout the world"—a tolerably numerous but disunited band.

Mrs. Stanton entertains no doubt of her mission and its value. "Lavater," she says, "predicted that a system of scientific physiognomy would be formulated within this century, and behold! it is here." Certainly nothing is so sure to win credence as a firm belief in one's own powers, and that first qualification for success is possessed by Mrs. Stanton. We know not if any of her ancestors originally hailed from the land of Burns, but we surmise that she has uttered the Scottish prayer, "Lord, send us a guid conceit o' oorsels," and that it has been amply fulfilled. The method by which she has attained her certainty is simple enough. Given a celebrity, declare that you see in his face the mark of his distinction. Point out anyone as John Jones, the philanthropist, and Mrs. Stanton at once exclaims, "Of course he is a philanthropist. He couldn't help it with that lower lip. Full, red, and moist, the lower lip always denotes benevolence." Suppose, however, you have made a mistake, and go on: "I beg your pardon. This is not John Jones, but his brother James Jones, the family scapegrace. He is a great grief, a constant scandal to his respectable relations. I needn't go into details, but he is about as bad as they make 'em; and what about his lower lip *now?*" Do you think that Mrs. Stanton is baffled? Not a bit of it. You don't know what is meant by a scientific system of physiognomy. The lower lip is thwarted by the eyes and hair, which belong to the lowest order of sensuality. If you only knew it, these are "polygamic eyes." "Have you a strawberry mark on your left arm?" asks Mr. Box of Mr. Cox. "No." "Then you *are* my long lost brother." This, or something like it, is the logic of the scientific system of physiognomy.

There are indeed some principles laid down, but they are simply confusing assertions. What, for example, can we make of this: "The kidney system creates or evolves *conscientiousness, integrity, morality.* The *width* of the chin, caused by width of its *bony structure,* denotes conscientiousness, as well as the strength and action of the kidney system. A narrow, retreating chin shows that the kidneys are narrow and small, a broad bony chin (if the eyes are well coloured) announces strong, large, or broad kidneys and relative breadth at the 'small of the back,' as it is termed. Taking into consideration that 75 per cent. of the human organism is composed of water, the importance of water as a fluid solvent of all the materials taken into the system, as well as its very important office as the carrier of all the materials through the veins and absorbent and secreting tubes to the several tissues involved in the human organism, it must be apparent that upon the power and activity of the fluid and kidney systems man depends very largely for the purity and integrity of his physical powers, hence of his moral nature."

We must apologise to our readers for the offensiveness of this piece of nonsense, and, indeed, if we were to quote too largely from some of Mrs. Stanton's pages we should be compelled to endless apologies, for they are largely defaced by prurient suggestion, sometimes, indeed, more than suggestion. The same may be said of one or two illustrations of embryonic types; but in justice we must admit that our author's pictures are the best, as well as the most amusing part of her book. They are mostly portraits of famous personages, with lines drawn to show what part of their face determines their most noted characteristics. When you know what your man is, it is easy enough to read his character in his countenance. We confess to being less impressed by Mrs. Stanton's clairvoyance in this matter than she seems to expect. Even when she informs us proudly that history confirms her estimate of the "godlike face" of Julius Cæsar, we are inclined to think that the good lady is putting the cart before the horse. History was there, and told its tale ere Mrs. Stanton breathed or dreamed of physiognomy. But there is no doubt that, whether or not they depend on the line of the nose and the setting of the eyes, the characteristics assigned by our scientific physiognomist to her characters are usually accurate enough. She has discovered that the most marked trait of Lady Burdett Coutts' character is benevolence, that Holbein the painter had an instinct for colour, that "Mother Byckerdyke," the Florence Nightingale of the American Civil War, is distinguished by sanativeness, while Miss Nightingale herself has much of the pneumatic faculty which takes cognisance of air and gases. Mrs. Stanton has even found out that the conspicuous facial and bodily sign of John L. Sullivan, the prize-fighter, is force. Far be it from us to contradict her regarding any one of these celebrities; but we cannot say that we think it very clever of her to find out what she has done. Apart from the nomenclature, indeed, we think that anyone among us could have guessed all that the system of scientific physiognomy has to tell us. When we come to a diplomatist whose face is full of caution, and a chemist whose nose betrays a capacity for analysis, we do not feel that a new scientific revelation has been vouchsafed us. It is all just what we expected. Alas! even scientific physiognomy brings us no new thing.

Sometimes, indeed, Mrs. Stanton is sufficiently amusing. Admirers of Miss Mary Anderson will be pleased to learn that the same sign is conspicuous in her face as in Shakespeare's. True, the sign is entitled "human nature," a characteristic which we had thought to be common to more than thirty million people in this island alone, and not infrequently met with elsewhere. But doubtless Mrs. Stanton means by human nature something different from what ordinary human nature does. She certainly sees more in it. But for ourselves, though we are glad to admit that man is made in the image of his Maker, however much defaced by sin and sorrow that image may be, we decline to follow the ingenious lady into the mysteries of conjugal eyes, sanative noses, and other wonders which she declares, but does not prove, to be writ large upon the countenance of man.

